# Development of the Preparation for Community‐Based Labor and Birth Instrument Centering Black Perspectives in the United States: A Participatory Adaptation

**DOI:** 10.1111/jmwh.70040

**Published:** 2025-10-28

**Authors:** Ashley Mitchell, Nikia Grayson, Patience A. Afulani, Kimberly Baltzell, Carrie Neerland, Alden Hooper Blair, Alexis Dunn Amore

**Affiliations:** ^1^ Institute for Global Health Sciences University of California, San Francisco San Francisco California; ^2^ CHOICES Center for Reproductive Health Memphis Tennessee; ^3^ School of Nursing University of California, San Francisco San Francisco California; ^4^ Departments of Obstetrics, Gynecology, and Reproductive Sciences and Epidemiology and Biostatistics University of California, San Francisco San Francisco California; ^5^ School of Nursing University of Minnesota Minneapolis Minnesota; ^6^ NYU Rory Meyers College of Nursing New York New York

**Keywords:** birth setting, freestanding birthing centers, health services, midwifery, perinatal care, survey

## Abstract

**Introduction:**

Community‐based birth supported by midwives and nurses is increasing in the United States amid stark racial disparities in maternal outcomes and worsening access to pregnancy care. Although studies examining prenatal confidence have shown that persons with higher confidence are more likely to give birth vaginally, reporting less pain, anxiety, and dissatisfaction, existing measurement tools have focused on hospital births. Accordingly, we adapted the previously validated Preparation for Labor and Birth (P‐LAB) instrument, which measures third‐trimester confidence for physiologic birth, for community‐based births, centering the perspectives of Black populations.

**Methods:**

Expert stakeholders (N = 5) including practicing midwives and maternal health researchers assessed the relevance and completeness of the P‐LAB. Following individual reviews, stakeholders adapted the tool during a group review session. Virtual cognitive interviews were then conducted with community stakeholders (N = 10), prenatal and newly postpartum persons, to test comprehensibility, informing further adaptation of P‐LAB items. Findings were summarized and analyzed using an abbreviated framework method. A subset of community stakeholders (N = 5) pretested the final instrument for redundancy and appropriateness.

**Results:**

The iterative adaptation process informed removal of irrelevant items (N = 6), further clarification of existing items (N = 12), and the generation of additional items (N = 7). The final instrument, the Preparation for Community‐Based Labor and Birth (P‐CLAB), is a 23‐item, Likert‐response survey. Expert stakeholder engagement resulted in replacing medication‐focused measures with items related to safety, dignity, and racial concordance while incorporating language aligning with the midwifery model of care. Community stakeholder engagement highlighted unclear items and opportunities to improve relevance.

**Discussion:**

In addition to promising utility for research, measuring prenatal confidence may equip midwives and nurses to further engage in person‐centered care by addressing maternal fears and empowering patients according to their specific needs. The participatory P‐CLAB adaptation enhances the instrument's utility and applicability to community‐based care settings.

## INTRODUCTION

National statistics for the United States suggest declining satisfaction with the quality of pregnancy care, with reports of mistreatment and disrespect increasing.^1^, ^2^, ^3^, ^4^ Concurrently, the United States continues to face the highest maternal mortality rate among high‐income countries with persistent and widening inequities, particularly for Black communities.^5^ Birthing persons—individuals with capacity for pregnancy—are increasingly seeking alternative care models outside of hospitals. Community births, supported by midwives in homes and freestanding birth centers, have trended upward since 2004, with more recent increases among non‐Hispanic Black birthing persons.^6^, ^7^, ^8^, ^9^ Multilevel racism and other forms of discrimination perpetuate poor health care experiences and outcomes for this group, informing care‐seeking behavior.^10^, ^11^, ^12^, ^13^, ^14^, ^15^ These intersecting experiences of worse outcomes and care, coupled with proliferating stories of reproductive inequities, perpetuate tokophobia (significant fear of childbirth). Tokophobia has been found to exacerbate stress, which is associated with higher preterm birth rates and decreased satisfaction, especially among Black populations.^13^, ^16^, ^17^, ^18^

Related to tokophobia is the concept of prenatal or childbirth confidence, which has much less robust evidence, despite associations suggesting that persons with higher confidence report less pain, anxiety, and dissatisfaction.^19^, ^20^, ^21^, ^22^ A recent concept analysis defined confidence for physiologic birth as a belief that it can be achieved, stemming both from personal values and from trusted support, information, and environments.^23^ Increased confidence has been associated with fewer unplanned interventions, including cesarean births.^22^ Furthermore, understanding the patient experience is a priority that has led to the development, testing, and use of a variety of instruments to inform improvements, particularly in clinical settings.^24^, ^25^ Measuring confidence as a key element of maternal experience may also inform person‐centered clinical and community‐based strategies.^26^, ^27^

Childbirth confidence research has prioritized hospital births and relied upon binary measures, limiting their utility.^26^ The recent development of the Preparation for Labor and Birth (P‐LAB) instrument by Neerland and colleagues (2020) was an important step toward more nuanced measurement of birthing confidence in the third trimester.^28^ Although this development is promising, with a content validity index of 0.95 and Cronbach's α coefficients of .74 to .93, the authors acknowledged key limitations in their validation sample of geographically Northern portions of the Midwestern United States and primarily White (>70%), college‐educated (>75%), and privately insured (80%) individuals.^28^ The instrument was also tested with participants planning hospital births, limiting its relevance to those seeking care in community‐based settings.^8^, ^29^, ^30^ Particularly as Black birthing people increasingly seek community‐based care, a more tailored approach is needed to address persistent disparities.^8^

The development of instruments for diverse, culturally specific groups would increase relevance by accounting for different needs and preferences. Persons who seek care at freestanding birth centers, for example, report valuing the midwifery model of care and the person‐centered culture and processes.^29^, ^30^ It is further perceived that their confidence is influenced by the unique physical space and continuity of care associated with out‐of‐hospital experiences.^30^ A targeted instrument intended for community‐based clinical use may allow for more timely evaluation of prenatal interventions intended to improve experiences or confidence. It may also assist with determining the most relevant birth setting for an individual given the differing resources available in a hospital versus community setting. Accordingly, the aim of this project was to adapt the P‐LAB instrument for clinical and research use with persons seeking community‐based care, centering the perspectives of Black birthing people. This article details our participatory approach to adaptation and the resulting instrument to measure childbirth confidence for physiologic birth in community‐based settings.

**QUICK POINTS**

✦ There is a growing preference for out‐of‐hospital, community‐based birth supported by midwives and nurses aligning with desires to mitigate discrimination and disparities in birth outcomes.
✦ Prenatal confidence has been shown to relate to improved maternal outcomes and experiences, although existing measures are limited.
✦ The development of the Preparation for Community‐Based Labor and Birth (P‐CLAB) is the result of an iterative, participatory approach informed by both intended implementers and participants.
✦ The P‐CLAB may guide patient‐provider dialogue related to patients’ choice of birth setting and overall preparedness for labor toward avoiding unplanned interventions and improving person‐centered care.

## METHODS

We followed DeVellis and Thorpe's widely applied instrument development guidelines to adapt the 22‐item Likert‐response P‐LAB instrument using an iterative approach between April and October 2024.^28^, ^31^ These guidelines were also used in the P‐LAB's original development.^28^ First, previously generated P‐LAB items were reviewed by expert stakeholders. Next, community stakeholders were engaged in cognitive interviewing, a specific technique in which an interviewer reviews each item with an intended participant, providing verbal probes to assess whether the intended meaning of each item aligns with the true meaning, and a subset engaged in piloting the revised instrument.^32^ Further steps involving psychometric evaluation are ongoing.

### Setting

The adaptation process was conducted in close partnership with CHOICES Memphis Center for Reproductive Health (CHOICES). CHOICES is a nonprofit community‐based clinic providing holistic, Black midwifery‐led care in Memphis, Tennessee. The center serves approximately 4000 individuals across more than 8500 appointments each year and offers full‐spectrum reproductive health care including gynecology, gender affirming care, fertility support, contraception, and more.^33^ CHOICES primarily serves Black (83.8%) and publicly insured (70.0%) patients (A. Blair, unpublished CHOICES Patient Data: 2017–2022), and its providers and staff are also predominately Black.^33^ Community‐based birth and perinatal services were introduced at CHOICES in 2017 aligning with its mission to provide comprehensive care from a Black feminist approach.^33^, ^34^ CHOICES has partnered with the Global Action in Nursing (GAIN) project at University of California, San Francisco (UCSF) since 2019 to measure efforts to improve Black maternal and neonatal health.^35^

An iterative, participatory approach was leveraged to center the perspectives of Black practitioners and birthing people; this was particularly important to counteract patterns of discrimination and exclusion.^3^, ^9^, ^12^, ^14^ Research has suggested that studies designed and implemented in collaboration with the target communities are critical to addressing the Black maternal health crisis, which further aligned with our goals.^36^, ^37^, ^38^ Accordingly, in both the expert and stakeholder reviews, Black scholars and women were prioritized.

#### Expert Stakeholder Review

The goal of the expert review process was to assess the content validity and completeness of the original P‐LAB instrument and to inform the adaptation of existing items for use in community settings, centering the perspectives of Black communities. We recruited a mix of 11 practicing midwives (including certified nurse‐midwives, a certified professional midwife, and a licensed midwife), reproductive equity researchers, members of the CHOICES Board of Directors, and other stakeholders via email. An initial pool was identified at the Midwifery is Public Health Conference held in Claremont, California (April 2023), with additional participants recruited through snowball sampling word‐of‐mouth recommendation. Of those recruited, 6 participants consented; 4 did not respond to the invitation and one declined due to time constraints. These experts were asked to complete a written assessment of the original P‐LAB instrument for relevance to community‐based birth and to Black‐identifying birthing persons (see Supporting Information: Appendix S1 for blank written assessment). Ultimately, 5 participants completed a written assessment in which they rated each item's relevance on a scale of 0 to 3 (0 = least relevant, 3 = most relevant).^39^ Participants also provided open‐ended comments and assessed the completeness of the preexisting instrument. The mean relevance score for each item and summarized comment themes informed the subsequent community stakeholder review. Given the anticipated uneven distribution of these results, the range of responses for each item was also reviewed.

Experts were then invited to attend a group review session via Zoom led by 3 of the authors (A.M., A.D.A., N.G.). Of the 6 invited, 3 participants joined and verbally consented to a 1.5‐hour session. The other experts reported scheduling conflicts. The discussion explored the items that had low average scores, or were neither unanimously “not relevant” nor “highly relevant”, as well as open‐ended comments from the written assessment. The conversation included both structured questions and unstructured dialogue, facilitated by a discussion guide (see Supporting Information: Appendix S2 for session guide). Instrument adaptation occurred in real‐time during the session, with proposed changes carefully reviewed and finalized afterward by all 3 facilitators. All expert stakeholders received a $50 gift card for their time and expertise.

#### Community Stakeholder Review

The adapted instrument was then tested through a community review process, which began with cognitive interviews to assess clarity through comprehension and ease of responding. We recruited current clients of CHOICES to participate in one‐on‐one cognitive interviews via Zoom with one researcher (A.M.). Clients who were at least 18 years of age and in their third trimester (≥28 weeks of pregnancy) of a singleton pregnancy or within 12 months postpartum were eligible to participate. CHOICES providers with routine access to patient data emailed 18 eligible clients with a study flyer (see Supporting Information: Figure S1 for flyer). Clients with recent visits were selected to prioritize those with current engagement in care. Eligible clients received 2 reminders before being determined unavailable to participate. Ten interested persons completed an eligibility screening and received a consent form via the secure web application, REDCap, prior to scheduling an interview via the scheduling software, Calendly. Following verbal consent, all 10 community stakeholders were engaged in recorded interviews to review instrument items using a detailed guide containing questions and probes (see Supporting Information: Appendix S3 for guide). All items of the adapted P‐LAB instrument were reviewed with each interviewee, toward establishing face validity, as well as to confirm relevance. Interviewees were also asked about the original response scale options (*strongly disagree*, *disagree*, *neutral*, *agree*, *strongly agree*). All community stakeholders received a $40 gift card for their time.

Detailed interview notes were analyzed using an expedited framework method.^40^ We leveraged stages 4 (framework development), 6 (charting), and 7 (interpretation) of Gale et al's (2013) approach to analyze findings in real‐time. Aligning with interview goals, we developed a working analytical framework with 3 a priori themes: (1) *comprehension*, (2) *ease of responding*, and (3) *perceived relationship to confidence*. Qualitative findings were then charted into the framework matrix in an Excel spreadsheet, organized by participant and item, immediately following each interview, and recordings were referenced as needed. Following all interviews, findings were reviewed by item and mapped in a second spreadsheet by theme. Mapped data were reviewed and interpreted by 3 authors (A.M., A.D.A., N.G.) to inform adaptation of items needing improvement.

The final adapted instrument was piloted with a subset of interviewees to assess for ease of responding to the adapted and new items, identify redundancy, and determine overall appropriateness. A subset of 8 interviewees was invited via email to participate in a pretest survey based on a diversity of described experiences. We sought a mix of nulliparous and multiparous participants, with varying described support systems, and both pregnant and postpartum persons. Eligible participants received 2 reminders before being determined unavailable to participate. Five community stakeholders consented and responded to the pretest survey using REDCap. The survey asked participants to respond to all adapted items and to then provide feedback on unclear items and ones that were difficult to answer. Participants were asked to respond as they would have during their third trimester of pregnancy, if they were no longer in that stage. Their responses to the specific items were not analyzed; however, feedback from their responses to 3 open‐ended questions about the survey items were reviewed. Participants received an additional $10 gift card for their time. Participant responses were exported to an Excel spreadsheet and reviewed for any suggestions to inform further item adaptation.

### Ethics

This project was designed and conducted in close partnership with leadership at CHOICES and approved by UCSF Human Research Protection Program (23‐40122).

#### Reflexivity Statement

The approach was conceptualized and implemented primarily by 3 authors (A.M., N.G., and A.D.A.), with iterative review and input from the larger team. Four authors bring midwifery or nursing experience (N.G., K.B., C.N., A.D.A.), and all have graduate training in public health. Three authors shared instrument design and adaptation experience, including one who designed the original P‐LAB (P.A.A., A.H.B., C.N.). All interpretation of results was conducted in partnership with 2 authors (N.G. and A.D.A.) who live and work in the study setting. We prioritized a Black feminist lens valuing participant autonomy and expertise, in alignment with CHOICES’ model of care.^33^

## RESULTS

**Figure 1:**
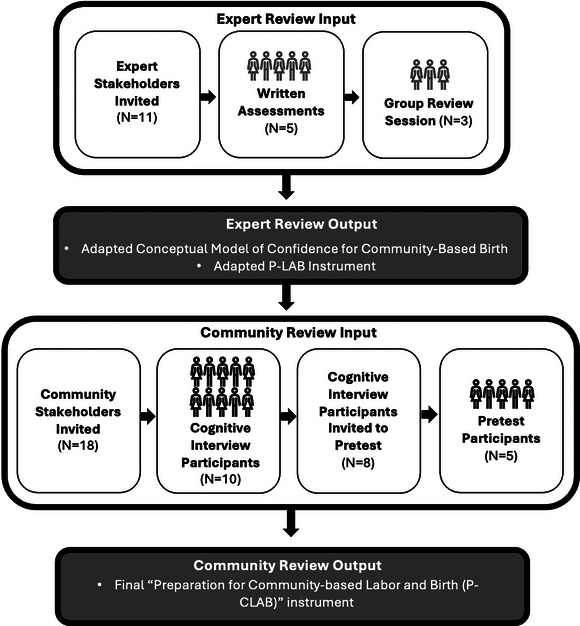
Instrument Adaptation Inputs and Outcomes Involving Expert and Community Reviews Abbreviation: P‐LAB, Preparation for Labor and Birth.

Our study resulted in the development of a 23‐item instrument, designed to measure confidence for physiologic birth in community‐based settings. To achieve this, we engaged in a participatory process to adapt the P‐LAB instrument for use with community‐based clients, particularly Black birthing populations (Figure 1). The 2‐step expert stakeholder review yielded an adapted conceptual model and instrument. The adapted version was then tested in a 2‐step community stakeholder review informing the final instrument.

### Expert Stakeholder Review

Experts’ ratings of the original instrument's relevance to confidence for community‐based birth and to pregnant care seekers at CHOICES varied by item (Table 1). Participating expert stakeholders held active roles as midwives, researchers, doulas, community organizers, advocates, and patient advisory board members, most spanning several roles at once. Overall, the expert‐perceived relevance to confidence (on a scale of 0‐3) for community‐based birth was slightly lower than the perceived relevance to CHOICES’ clients (2.69 vs 2.62, respectively).

**Table 1:** Expert Stakeholder's Perceived Relevance (0 = Not Relevant to 3 = Highly Relevant)^a^ of the Original Preparation for Labor and Birth Instrument by Item

		Relevance to Confidence for Community‐Based Birth						Relevance to CHOICES’ Clients					
		Reviewer						Reviewer					
Item no.	Original P‐LAB Item	A	B	C	D	E	Mean	A	B	C	D	E	Mean
1	I feel comfortable with where I will give birth	3	2	1	3	3	2.4	3	2	2	3	3	2.6
2	I will have the support that I need from my partner, doula, or other support person(s) in labor	3	2	3	3	3	2.8	3	2	3	3	3	2.8
3	I am confident in my body's ability to labor and birth	3	3	3	3	3	3	3	3	3	3	3	3
4	It is important to me to use medication in labor for pain relief (for example: IV medications or epidural anesthesia)	3	1	2	1	2	1.8	3	3	2	1	2	2.2
5	I trust that my prenatal care provider(s) will respect my preferences in labor	3	3	3	3	3	3	1	3	3	3	3	2.6
6	When I think about labor and birth, I am fearful	2	3	2	3	3	2.6	2	2	2	3	3	2.4
7	I have sought out childbirth information from multiple sources	2	3	1	3	3	2.4	2	3	1	3	3	2.4
8	I feel prepared to give birth without the use of pain medication (for example: IV medications or epidural anesthesia)	2	3	2	3	3	2.6	2	2	2	3	3	2.4
9	Negative birth stories from others have made me more fearful about birth	3	3	2	2	2	2.4	3	3	2	2	2	2.4
10	I am excited about experiencing childbirth	2	‐	2	2	2	2	2	‐	2	2	2	2
11	I plan to give birth with the use of pain medication (for example: IV pain medication or epidural anesthesia)	2	‐	3	2	3	2.5	2	‐	3	1	3	2.25
12	My support person(s) (partner, doula, or other) is/are supportive of my childbirth preferences	3	‐	3	3	2	2.75	3	‐	3	3	2	2.75
13	It is important to me to experience childbirth without any pain medication (for example: IV pain medication or epidural anesthesia)	2	2	2	3	3	2.4	2	2	2	3	3	2.4
14	My prenatal care provider(s) discuss(es) options and choices with me	3	2	3	3	3	2.8	3	3	3	3	3	3
15	I do not feel that I have enough information about the childbirth process	3	3	2	3	3	2.8	3	3	2	3	3	2.8
16	My prenatal care provider communicates with me in an honest and respectful manner	3	2	2	3	3	2.6	3	3	2	3	3	2.8
17	I am receiving the right amount of emotional support from my partner, doula, or other labor support person(s)	3	2	1	3	2	2.2	3	3	1	3	2	2.4
18	I am confident that I will be able to cope with labor pain	3	3	2	3	3	2.8	3	3	2	3	3	2.8
19	I know my own preferences for labor and birth	3	1	3	3	3	2.6	3	3	3	3	3	3
20	I wish I were better prepared for labor and birth	2	3	2	3	3	2.6	2	3	2	3	3	2.6
21	My prenatal care provider addresses my needs during prenatal visits	3	3	3	3	3	3	3	3	3	3	3	3
22	My birth will take place in a calm, supportive environment	3	3	3	3	3	3	3	3	3	3	3	3

Abbreviations: IV, intravenous; P‐LAB, Preparation for Labor and Birth.

a For each, reviewers could select: 0, Not relevant; 1, Somewhat relevant; 2, Quite relevant; or 3, Highly relevant.

Broadly, comments suggested incorporating additional domains such as racial congruence and measures of respect. One reviewer wrote: “With recent social media attention to disparities in Black Birth this fear is heightened in our communities” (Expert Stakeholder A). Factors like discrimination, bias, and racism were described repeatedly as important to consider; another person wrote: “It would be important to include measures that capture respect and believability during the birth process” (Expert Stakeholder B). Comments also highlighted the nuances with community‐based birth that were not yet captured, suggesting that “It may also be important to ask a question pertaining to them visualizing the birth they want” (Expert Stakeholder D). Both the item scores and comments were discussed during the group review session.

Three expert stakeholders attended the virtual group review session. Items deemed less relevant (average rating of 2.5 or less) were discussed along with items with discordant ratings (ie, a score of 1 vs 3 by different reviewers). As a result of the discussion, 6 items were dropped (4, 8, 11, 13, 15, 17); most of these items included components that were not relevant to community‐based birth (ie, epidural anesthesia). Additionally, the language of 8 items (1, 2, 6, 7, 9, 10, 12, 18) was slightly revised to capture priorities of diverse, community‐based clients. For instance, the original item “I have sought out childbirth information from multiple sources” was adapted to “I have been able to find childbirth information that aligned with my priorities,” as experts agreed a trusted source is often more important than multiple sources for these clients. Likewise, clarifying text was added to items including the first one, “I feel comfortable with where I will give birth,” to specify the adapted instrument's intent to engage respondents intending to birth at freestanding birth centers or their home. Finally, 6 new items were drafted as reviewers recognized gaps including acknowledgments of safety, dignity, and racial identity as factors they perceive to influence confidence for community‐based birth, particularly among Black birthing persons.

The conceptualization of confidence to achieve physiologic childbirth used in the original instrument was also further adapted as a result of the expert stakeholder review.^28^ The broad concepts including the influence of beliefs about labor, the support a person receives, and the birthing environment remained.^28^ The “Planned Use of Pain Medication” factor was dropped, however, and language was simplified to underscore the salient values of community‐based birth (see Supporting Information: Figure S2 for conceptual model). The adapted, hypothesized conceptual model includes 4 factors: (1) support persons, (2) intrinsic preparation, (3) extrinsic influences, and (4) space/place (Table 2). These factors were chosen given their alignment with prior research suggesting that the birth center model contributes to confidence through the culture (space/place), midwifery care model (support persons), internal influences (intrinsic preparation), and outside influences (external influences).^29^ Each of the adapted items were assigned to one of these factors.

**Table 2:** Hypothesized Conceptual Factors of Confidence to Achieve Physiologic Childbirth Adapted for Community‐Based Care Settings

Original P‐LAB Factor	Adapted Factor^a^	Brief Definition
Beliefs About labor	Intrinsic preparation	Personal preferences, feelings, and perceptions
Relationship with care provider and supportive birth environment	Space/place	Comfort and access to desired labor supports within the environment the respondent plans to labor and birth
Labor support	Support persons	Involvement of prenatal care providers as well as any other person the respondent may involve in their pregnancy or labor
Planned use of pain medication	Extrinsic influences	Ability to access relevant resources as well as input from others relating to the respondent's pregnancy or birth

Abbreviation: P‐LAB, Preparation for Labor and Birth.

a Adapted factors were informed by thematic findings of Neerland and Skalisky (2022).^29^

### Community Stakeholder Review

Responses from interviewees informed adaptations of both existing and new items.

#### Review of Existing Items

Findings relevant to each of the 3 a priori themes, *comprehension*, *ease of response*, and *perceived relationship to confidence*, were charted for each participant by item. Summaries were then reviewed item by item and mapped by theme (see Supporting Information: Table S1 for charting).

### Comprehension

Items for which comprehension was challenging or discordant for interviewees were revised. For example, the text in the parenthesis that followed the previously described item “I have been able to find childbirth information that aligned with my priorities (ie, cultural relevance, trusted source, accessibility, etc.)” was found to be jargon and meant different things to different interviewees. Accordingly, this item was updated to “When I have had questions, I have been able to find childbirth information that I found beneficial.”

### Ease of Response

Likewise, items that were difficult for several interviewees to answer were revisited. For instance, several participants struggled to respond to the original item “My prenatal care provider(s) discuss(es) options and choices with me” and suggested they were not sure what “options and choices” were referring to. This item was adapted to “My prenatal care provider(s) are giving me what I need to feel prepared for labor and birth (ie, educating me on my options/choices).”

### Perceived Relation to Confidence

Finally, items for which the perceived relevance to prenatal confidence was weak were reconsidered as well. For example, the adapted item “At this point when I think about labor and birth, I am fearful” led to many nuanced conversations in which interviewees shared they could be both fearful and confident. This item was ultimately changed to “At this point when I think about labor and birth, I am more confident than fearful.”

#### Review of New Items

Special consideration was paid to the 6 new items developed during the expert stakeholder review process to ensure they were perceived to be valid and relevant. We were specifically interested to verify that they addressed the perceived gaps of the original P‐LAB for this population including *safety*, *dignity*, and *racial identity*.

### Safety

When discussing drafted items related to safety, community stakeholders unanimously agreed this impacted their confidence. One interviewee shared “my mental and physical being are 100% being looked after … it definitely brings confidence up” (Interviewee 5). Others agreed and equated feeling safe with feeling comfortable and protected. In response to dignity‐related items, interviewees shared similar strong agreement.

### Dignity

While reviewing items about respect and believability, one person explained “Like [as a prenatal client at CHOICES] I'm not just another person, like I'm an actual human … I'm respected, understood, heard” (Interviewee 1). Other interviewees described that they felt respected in their care; one person said, “I wasn't just a number or checking things off of a list” (Interviewee 6), while another affirmed the care they received “made [them] feel like they would be there for me during labor and I wouldn't have to question [provider's] intentions” (Interviewee 10).

### Racial Identity

Finally, when reviewing the item related to racial identity, participants agreed it was important to include. Many interviewees described the power and relevance of being listened to as Black clients, with statements like “I see me” (Interviewee 8), when asked to elaborate on why race mattered. One interviewee explained, “…knowing that I'm the race I am, I didn't feel like I had to fight for [care] there” (Interviewee 4). Several participants referenced the abysmal Black maternal morbidity and mortality rates in the United States, emphasizing things such as “…when I found [CHOICES], and I saw that they were all Black, I felt like it would be more relatable because I've seen a lot of women not be listened to, Black women … especially while they're pregnant” (Interviewee 10).

In addition to retaining the 6 previously drafted items, one additional item was added following cognitive interviews, “I have people who listen to and believe me about my experiences in pregnancy,” given the frequency at which interviewees described the importance of these things while discussing other items.

#### Pretest Summary

All participants agreed that no question was difficult to answer. In response to “Were there questions that felt repetitive?” one participant wrote “questions concerning being prepared for labor” whereas all other participants wrote “None.” Given the instrument's intended focus on preparation for labor, no changes were made in response to this comment. Similarly, all participants wrote “None” when asked if there was anything else we should know. Overall, no changes were made to the items following the pretest.

### Adapted Instrument

The final instrument, “Preparation for Community‐Based Labor and Birth (P‐CLAB),” contained 23 items (Table 3). Of the original P‐LAB instrument's items, 4 remained the same (new prompt numbers 3, 4, 15, 16) whereas all other items were adapted in some way, dropped, or replaced by new items determined to be more relevant for community‐based birth, particularly among Black communities (see Supporting Information: Table S2 for item evolution).

**Table 3:** Final Preparation for Community‐Based Labor and Birth Prompts and Response Options^a^

No.	Prompt
1	I feel comfortable with planning to birth in a community setting (ie, not at a hospital)
2	I believe I will have the support that I need from my partner, doula, and/or other nonmedical support person(s) in labor
3	I am confident in my body's ability to labor and birth
4	I trust that my prenatal care provider(s) will respect my preferences in labor
5	At this point when I think about labor and birth, I am more confident than fearful
6	When I have had questions, I have been able to find childbirth information that I found beneficial
7	Negative comments about my birth plan, my own past experiences, and/or stories from others have made me more fearful
8	I feel ready for birth (or believe I will be by the time the baby comes)
9	Positive comments about my birth plan, my own past experiences, and/or stories from others have inspired confidence for me
10	My prenatal care provider(s) are giving me what I need to feel prepared for labor and birth (ie, educating me on my options/choices)
11	My prenatal care provider communicates honestly with me
12	I believe that I will have what I need to cope with the pain of labor and birth (ie, aromatherapy, a birthing tub, etc.)
13	I know my own preferences for labor and birth—even if my preference is going with the flow
14	I am worried that when labor begins, I won't be prepared for it
15	My prenatal care provider addresses my needs during prenatal visits
16	My birth will take place in a calm, supportive environment
17	Given my racial identity, I feel comfortable with the providers with whom I receive prenatal care
18	When I visualize the birth that I want, I believe I will have the tools that I need to accomplish it (ie, music, knowledge of different positions to labor/birth, breathing techniques, etc.)
19	I feel safe in the physical space that I plan to birth
20	I feel protected by my prenatal provider
21	My self‐talk as I prepare for birth is mostly positive
22	I sense that my prenatal provider cares about me as a person—or will make every effort to do so by the time I birth
23	I have people who listen to and believe me about my experiences in pregnancy

a The instructions were as follows: “Please answer the following questions about your own feelings of preparedness and confidence for labor and birth. Try to answer honestly based on how you feel at this stage in your pregnancy.” Response options for all prompts include “Strongly Disagree,” “Disagree,” “Neutral,” “Agree,” “Strongly Agree”; prompt 4 also has the option “Not Applicable” for respondents who do not perceive to have preferences.

## DISCUSSION

Our iterative, community‐engaged approach resulted in a 23‐item adaptation of the P‐LAB instrument, the P‐CLAB. Broadly, the adapted version aligns with the original P‐LAB instrument, with several items remaining the same and the shared overarching goal of measuring third‐trimester confidence to inform the need for targeted interventions.^28^ Additionally, a 4‐factor hypothesized conceptual model was maintained and adapted. This instrument can contribute to the development and use of perinatal interventions that enhance confidence and inform quality of care.

Many instruments designed to measure prenatal confidence to date have not been developed for use by health care providers and instead have been intended for research purposes.^26^ The original P‐LAB instrument was among the first with the potential for clinical utility, involving both providers and patients in its development.^28^ This adaptation underscored that including a provider perspective, from development forward, increases the likelihood that timely intervention can occur for the patients with whom it is used. Likewise, the involvement of Black‐identifying individuals as expert and community stakeholders ensured the inclusion of relevant items by centering Black perspectives. Values including safety, dignity, and racial identity were emphasized throughout the adaptation in alignment with prior suggestions that autonomy and respect are prioritized in community birth settings.^8^, ^9^, ^41^ Our processes affirmed the feasibility of community‐engaged instrument development as well as the value of tailoring items to diverse participant groups.^42^

The multistep engagement of both expert and community stakeholder groups also ensured careful assessment of the instrument's relevance to prenatal confidence. Existing instruments have largely prioritized self‐efficacy, which has been conceptualized as successfully executing a task to obtain a specific outcome, versus the broader concept of confidence as a general sense of self‐assuredness applicable to dynamic circumstances like that of childbirth.^23^, ^43^

Similar to the original P‐LAB instrument, the P‐CLAB may be used to support decision‐making regarding birth setting. This is especially important in states where the integration of midwives into the broader birth care system is lagging, particularly among southern states, including Tennessee.^44^, ^45^ In these settings, transfers of care for perinatal emergencies, additional pain management, or other reasons can be challenging due to a lack of respectful interprofessional collaboration.^45^ In addition to the geographic location, there are challenges with care transfers from birth centers to hospitals, further exacerbating unnecessary risks for birthing persons.^44^, ^45^ Although improved collaboration between settings is critical to long‐term change, increased upstream patient‐provider dialogue surrounding birth setting may mitigate some of the negative experiences in the meantime. Furthermore, although early analysis of the P‐LAB suggested extra care be taken with nulliparous persons, more research is needed to explore whether persons with prior birth experience consistently have higher confidence in more racially diverse and/or community‐based settings.^46^

### Strengths and Limitations

Our participatory adaptation approach was a key study strength. The adapted instrument is promising for use as one of many conversational tools to assess and bolster prenatal confidence. Still, we recognize we may have missed the inclusion of important community subsets given that participants for the cognitive interviews and pretests were selected from a single birth center, whose patients are relatively homogenous related to race and sociocultural status. Our reviews also had relatively small sample sizes. Expert reviewers were particularly challenging to recruit given their competing responsibilities. Even when a second virtual group review session was offered, only one additional participant had availability. Still, we believe the involvement of 2 experts (N.G., A.D.A.) throughout mitigated some of these limitations. Additionally, the use of a subset of interviewees for the pretest may have been influenced by the reality that these individuals already provided feedback about these topics. On the other hand, previously engaged participants may have been more likely to take their review task more seriously.

### Research Implications

The P‐CLAB is now being administered at multiple Black‐led birth centers in the United States, and psychometric analysis is planned toward further validation. Continuing DeVellis and Thorpe's guidelines, we plan to conduct factor analysis, which may reduce items or refine hypothesized factors.^31^ Still, the instrument may be informative, even without validation, as researchers of prior group‐specific instruments have recommended including items recommended by the community despite poor psychometric performance.^42^ This aligns with a growing recognition of the value of community engagement in the design and development of instruments and research in general.^47^, ^48^

Future studies of prenatal confidence within the community birth setting should explore perspectives of broader, racially diverse care seekers to expand the instrument's utility. Additionally, although we did not receive comments about the number of items during the adaptation process, participant burden could occur with the length of the scale. It is possible this will be addressed during psychometric analysis and further validation efforts. Finally, additional work must examine effective interventions and guides for providers to support the interpretation of responses and scores.

### Clinical Implications

The adapted instrument has the potential to facilitate targeted patient‐provider conversations with prenatal clients to promote understanding about the factors influencing childbirth confidence. Intervening during the prenatal period may mitigate fears related to birth and improve patient experiences or outcomes. On the other end of the spectrum, it could also support informed dialogue for individuals for whom a hospital setting may be most appropriate or preferred. Each of these opportunities may prevent transfers during labor that are not medically indicated. Relevant measurement that highlights opportunities to increase positive experiences may also contribute to improved quality of care, known to improve maternal and infant outcomes.^22^

## CONCLUSION

The P‐CLAB has the potential to inform patient‐provider interactions and prenatal interventions that improve childbirth experiences among populations at greater risk for mistreatment, discrimination, and poor outcomes across the perinatal period. Our community‐engaged approach yielded a promising adaptation ensuring that those most affected (Black birthing persons) were represented in creating solutions to improve patient‐centered approaches to perinatal care.^37^, ^38^ The ability to measure, and engage pregnant individuals about, confidence may mitigate unplanned interventions and inform person‐centered care.^22^, ^26^, ^27^ Tailored instruments including the P‐CLAB may provide insight to improve quality of care for populations most at risk for persistent maternal disparities in the United States.

## CONFLICT OF INTEREST

The authors have no conflicts of interest to disclose.

## Supporting information




**Appendix S1**. Key Stakeholder Review Written Assessment
**Appendix S2**. Key Stakeholder Group Review Session Guide
**Figure S1**. Recruitment Flyer for Cognitive Interviews
**Appendix S3**. Cognitive Interview Guide
**Figure S2**. Adapted Conceptual Model of Confidence for Community‐Based Birth
**Table S1**. Cognitive Interview Findings Summaries Mapped By A Priori Themes
**Table S2**. Instrument Item Evolution from Original Items to Final Adapted Item Following Expert and Community Stakeholder Review Processes

Supporting Information

## References

[1] Global Women's Health Index: United States. Hologic website . 2022. Accessed October 27, 2023. https://hologic.womenshealthindex.com/index‐rankings/united‐states

[2] Mohamoud YA , Cassidy E , Fuchs E , et al. Vital Signs: Maternity Care Experiences — United States, April 2023. MMWR Morb Mortal Wkly Rep. 2023;72(35):961‐967. doi:10.15585/mmwr.mm7235e1 37651304

[3] Odems DS , Czaja E , Vedam S , Evans N , Saltzman B , Scott KA . “It seemed like she just wanted me to suffer”: acts of obstetric racism and birthing rights violations against Black women. SSM Qual Res Health. 2024;6:100479. doi:10.1016/j.ssmqr.2024.100479

[4] Cayama MR , Vamos CA , Harris NL , Logan RG , Howard A , Daley EM . Respectful maternity care in the United States: a scoping review of the research and birthing people's experiences. J Midwifery Womens Health. 2025;70(2):212‐222. doi:10.1111/jmwh.13729 39812176

[5] Gunja MZ , Gumas ED , Williams RD II . The U.S. maternal mortality crisis continues to worsen: an international comparison. The Commonwealth Fund. December 1, 2022. doi:10.26099/8vem-fc65

[6] MacDorman MF , Barnard‐Mayers R , Declercq E . United States community births increased by 20% from 2019 to 2020. Birth. 2022;49(3):559‐568. doi:10.1111/birt.12627 35218065

[7] MacDorman MF , Declercq E . Trends and state variations in out‐of‐hospital births in the United States, 2004‐2017. Birth. 2019;46(2):279‐288. doi:10.1111/birt.12411 30537156 PMC6642827

[8] Sakala C , Hernandez‐Cancio S , Wei R . Improving Our Maternity Care Now Through Community Birth Settings | National Partnership for Women & Families . National Partnership for Women & Families; 2022. Accessed August 11, 2024. https://nationalpartnership.org/report/community‐birth‐settings/ 10.1891/JPE-2022-0015 PMC9584101 36277227

[9] Karbeah J , Hardeman R , Katz N , Orionzi D , Kozhimannil KB . From a place of love: the experiences of birthing in a Black‐owned culturally‐centered community birth center. J Health Disparities Res Pract. 2022;15(2):47‐60. PMC10237589 37275571

[10] Crear‐Perry J , Correa‐de‐Araujo R , Lewis Johnson T , McLemore MR , Neilson E , Wallace M . Social and structural determinants of health inequities in maternal health. J Womens Health. 2021;30(2):230‐235. doi:10.1089/jwh.2020.8882 PMC8020519 33181043

[11] Burris HH , Hacker MR . Birth outcome racial disparities: a result of intersecting social and environmental factors. Semin Perinatol. 2017;41(6):360‐366. doi:10.1053/j.semperi.2017.07.002 28818300 PMC5657505

[12] Vedam S , Stoll K , Taiwo TK , et al. The Giving Voice to Mothers study: inequity and mistreatment during pregnancy and childbirth in the United States. Reprod Health. 2019;16(1):1‐18. doi:10.1186/s12978-019-0729-2 31182118 PMC6558766

[13] Braveman P , Heck K , Egerter S , et al. Worry about racial discrimination: a missing piece of the puzzle of Black‐White disparities in preterm birth? PloS One. 2017;12(10):e0186151. doi:10.1371/journal.pone.0186151 29020025 PMC5636124

[14] Anyiam S , Woo J , Spencer B . Listening to Black women's perspectives of birth centers and midwifery care: advocacy, protection, and empowerment. J Midwifery Womens Health. 2024;69(5):653‐662. doi:10.1111/jmwh.13635 38689459

[15] Odems DS , Czaja E , Vedam S , Evans N , Saltzman B , Scott KA . Manifestations of anti‐Black racism and worry about pregnancy and birthing while Black: a cross‐sectional secondary analysis of Giving Voice to Mothers. J Racial Ethn Health Disparities. Published online May 6, 2025. doi:10.1007/s40615-025-02461-2 PMC13346305 40327291

[16] Roosevelt LK , Low LK . Understanding fear of childbirth among underrepresented populations in the United States. J Health Psychol. 2021;26(14):2811‐2821. doi:10.1177/1359105320922306 32538163

[17] Hawkins S, Lam S , Verani S . Birthing justice: Black doulas on racism, fear, and reproductive health in the BLM era. The Oaklandside. September 4, 2020. Accessed September 19, 2023. http://oaklandside.org/2020/09/04/birthing‐justice‐black‐doulas‐on‐racism‐fear‐and‐reproductive‐health‐in‐the‐blm‐era/

[18] Thayer ZM , Geisel‐Zamora SA , Uwizeye G , Gildner TE . Childbirth fear in the USA during the COVID‐19 pandemic: key predictors and associated birth outcomes. Evol Med Public Health. 2023;11(1):101‐111. doi:10.1093/emph/eoad006 37090221 PMC10114526

[19] Crowe K , von Baeyer C . Predictors of a positive childbirth experience. Birth Berkeley Calif. 1989;16(2):59‐63. doi:10.1111/j.1523-536x.1989.tb00862.x 2757721

[20] Lowe NK . Maternal confidence in coping with labor. A self‐efficacy concept. J Obstet Gynecol Neonatal Nurs. 1991;20(6):457‐463. doi:10.1111/j.1552-6909.1991.tb01711.x 1757830

[21] Browne J , O'Brien M , Taylor J , Bowman R , Davis D . “You've got it within you”: the political act of keeping a wellness focus in the antenatal time. Midwifery. 2014;30(4):420‐426. doi:10.1016/j.midw.2013.04.003 23747292

[22] Attanasio LB , McPherson ME , Kozhimannil KB . Confidence and positive childbirth experiences in U.S. hospitals: a mixed methods analysis. Matern Child Health J. 2014;18(5):1280‐1290. doi:10.1007/s10995-013-1363-1 24072597 PMC3966989

[23] Neerland CE . Maternal confidence for physiologic childbirth: a concept analysis. J Midwifery Womens Health. 2018;63(4):425‐435. doi:10.1111/jmwh.12719 29874705

[24] Macpherson I , Roqué‐Sánchez MV , Legget B Finola O , Fuertes F , Segarra I . A systematic review of the relationship factor between women and health professionals within the multivariant analysis of maternal satisfaction. Midwifery. 2016;41:68‐78. doi:10.1016/j.midw.2016.08.003 27551856

[25] Larrabee Sonderlund A , Schoenthaler A , Thilsing T . The association between maternal experiences of interpersonal discrimination and adverse birth outcomes: a systematic review of the evidence. Int J Environ Res Public Health. 2021;18(4):1465. doi:10.3390/ijerph18041465 33557227 PMC7913961

[26] Avery MD , Saftner MA , Larson B , Weinfurter EV . A systematic review of maternal confidence for physiologic birth: characteristics of prenatal care and confidence measurement. J Midwifery Womens Health. 2014;59(6):586‐595. doi:10.1111/jmwh.12269 25533706

[27] Avery MD , Neerland CE , Saftner MA . Women's perceptions of prenatal influences on maternal confidence for physiologic birth. J Midwifery Womens Health. 2019;64(2):201‐208. doi:10.1111/jmwh.12897 30334320

[28] Neerland CE , Avery MD , Looman WS , Saftner MA , Rockwood TH , Gurvich OV . Development and testing of the preparation for labor and birth instrument. J Obstet Gynecol Neonatal Nurs. 2020;49(2):200‐211. doi:10.1016/j.jogn.2019.12.006 32035974

[29] Neerland CE , Skalisky AE . A qualitative study of US women's perspectives on confidence for physiologic birth in the birth center model of prenatal care. J Midwifery Womens Health. 2022;67(4):435‐441. doi:10.1111/jmwh.13349 35246924

[30] Neerland CE , Delkoski SL , Skalisky AE , Avery MD . Prenatal care in US birth centers: midwives’ perceptions of contributors to birthing People's confidence in physiologic birth. Birth. 2023;50(3):535‐545. doi:10.1111/birt.12676 36226921

[31] DeVellis RF , Thorpe CT . Scale Development: Theory and Applications. SAGE Publications; 2021.

[32] Hibben Kristen Cibelli de Jong Julie . Cognitive Interviewing. In: Guidelines for Best Practice in Cross‐Cultural Surveys. Survey Research Center, Institute for Social Research, University of Michigan; 2016. Accessed July 31, 2025. https://ccsg.isr.umich.edu/chapters/pretesting/cognitive‐interviewing/

[33] Grayson N , Quinones N , Oseguera T . A model of true CHOICES: learnings from a comprehensive sexual and reproductive health clinic in Tennessee that provides abortions and opened the city's first birth center. J Midwifery Womens Health. 2022;67(6):689‐695. doi:10.1111/jmwh.13448 36471539

[34] Ross LJ . Reproductive justice as intersectional feminist activism. Souls. 2017;19(3):286‐314. doi:10.1080/10999949.2017.1389634

[35] Global Action in Nursing (GAIN) . University of California San Francisco website. 2025. Accessed August 18, 2023. https://gainproject.ucsf.edu/global‐action‐nursing

[36] Altman MR , McLemore MR , Oseguera T , Lyndon A , Franck LS . Listening to Women: recommendations from women of color to improve experiences in pregnancy and birth care. J Midwifery Womens Health. 2020;65(4):466‐473. doi:10.1111/jmwh.13102 32558179

[37] Hernandez N , Guillaume D , Clarke L , et al. Centering cultural approaches in community‐based participatory research to address the Black maternal health crisis. AJPM Focus. 2025;4(6):100402. doi:10.1016/j.focus.2025.100402 41078995 PMC12509760

[38] Black Mamas Matter Alliance . *Black Maternal Health Research Re‐Envisioned: Best Practices for the Conduct of Research With, For, and By Black Mamas* . Black Mamas Matter Alliance; 2019. Accessed August 5, 2025. https://blackmamasmatter.org/wp‐content/uploads/2022/10/BMMA_RP_Report_1026.pdf

[39] Yusoff MSB . ABC of content validation and content validity index calculation. Educ Med J. 2019;11(2):49‐54. doi:10.21315/eimj2019.11.2.6

[40] Gale NK , Heath G , Cameron E , Rashid S , Redwood S . Using the framework method for the analysis of qualitative data in multi‐disciplinary health research. BMC Med Res Methodol. 2013;13:117. doi:10.1186/1471-2288-13-117 24047204 PMC3848812

[41] Almanza JI , Karbeah J’ , Tessier KM , et al. The impact of culturally‐centered care on peripartum experiences of autonomy and respect in community birth centers: a comparative study. Matern Child Health J. 2022;26(4):895‐904. doi:10.1007/s10995-021-03245-w 34817759 PMC9012707

[42] Afulani PA , Altman MR , Castillo E , et al. Development of the person‐centered prenatal care scale for people of color. Am J Obstet Gynecol. 2021;225(4):427.e1‐427.e13. doi:10.1016/j.ajog.2021.04.216 33862014

[43] Bandura A . Self‐Efficacy: The Exercise of Control. Macmillan; 1997.

[44] Vedam S , Stoll K , MacDorman M , et al. Mapping integration of midwives across the United States: impact on access, equity, and outcomes. PLoS One. 2018;13(2):e0192523. doi:10.1371/journal.pone.0192523 29466389 PMC5821332

[45] Caughey AB , Cheyney M . Home and birth center birth in the United States: time for greater collaboration across models of care. Obstet Gynecol. 2019;133(5):1033. doi:10.1097/AOG.0000000000003215 31022111

[46] Neerland CE , Avery MD , Saftner MA , Gurvich OV . Maternal confidence for physiologic birth: associated prenatal characteristics and outcomes. Midwifery. 2019;77:110‐116. doi:10.1016/j.midw.2019.07.004 31319365

[47] Kelley A , Piccione C , Fisher A , Matt K , Andreini M , Bingham D . Survey development: community involvement in the design and implementation process. J Public Health Manag Pract. 2019;25:S77‐S83. doi:10.1097/PHH.0000000000001016 31348193 PMC6662621

[48] Moczygemba LR , Singh RL , Baffoe JO . Research and scholarly methods: community‐engaged research. J Am Coll Clin Pharm. 2023;6(12):1366‐1373. doi:10.1002/jac5.1881

