# Supplementary material for: Development of the Preparation for Community‐Based Labor and Birth Instrument Centering Black Perspectives in the United States: A Participatory Adaptation
Source: J Midwifery Womens Health. 2025 Oct 28;71(1):76–86. doi: 10.1111/jmwh.70040 (PMC12914615; doi:10.1111/jmwh.70040)
Supplement: Supplementary file 1 — Appendix S1. Key Stakeholder Review Written Assessment Appendix S2. Key Stakeholder Group Review Session Guide Figure S1. Recruitment Flyer for Cognitive Interviews Appendix S3. Cognitive Interview Guide Figure S2. Adapted Conceptual Model of Confidence for Community‐Based Birth Table S1. Cognitive Interview Findings Summaries Mapped By A Priori Themes Table S2. Instrument Item Evolution from Original Items to Final Adapted Item Following Expert and Community Stakeholder Review Processes [file JMWH-71-76-s002.docx]

**Supporting Information**

1. Supporting Appendix S1: Key Stakeholder Review Written Assessment (pp. 1–7)
2. Supporting Appendix S2: Key Stakeholder Group Review Session Guide (pp. 8–12)
3. Supporting Figure S1: Recruitment Flyer for Cognitive Interviews (p. 13)
4. Supporting Appendix S3: Cognitive Interview Guide (pp. 14–23)
5. Supporting Figure S2: Adapted Conceptual Model of Confidence for Community-Based Birth (p. 24)
6. Supporting Table S1: Cognitive Interview Findings Summaries Mapped By A Priori Themes (pp. 25–28)
7. Supporting Table S2: Instrument Item Evolution from Original Items to Final Adapted Item Following Expert and Community Stakeholder Review Processes (pp.29–32)

**Supporting Appendix S1: Key Stakeholder Review Written Assessment**

**Preparation for Labor and Birth (P-Lab) Instrument Adaptation**

*Key Stakeholder Review*

*Return to Ashley Mitchell (*[*ashley.mitchell@ucsf.edu*](mailto:ashley.mitchell@ucsf.edu)*) by April 30^th^, 2024.*

**About the Study**

This study aims to adapt a tool to measure individuals’ confidence for childbirth in a community-based setting. Studies suggest that individuals with higher childbirth confidence report less pain, anxiety, and dissatisfaction. Given the inverse relationship between confidence and fear, and prior associations between maternal stress and preterm birth, it is also possible that confidence could impact gestation length.^[[1]](#footnote-1)^ There is a need for more nuanced study of confidence surrounding childbirth. Specifically, we will be adapting the *Preparation for Labor and Birth (P-LAB)* instrument (described below). This instrument was developed and tested in 2020. While promising and informed by midwifery perspectives, the tool is limited by its testing sample of Northern and primarily white (>70%), college educated (>75%), married (83%), and privately insured (80%) individuals.^[[2]](#footnote-2)^ We intend to ensure its content is meaningful for birthing clients at CHOICES Memphis Center for Reproductive Health, 84% of whom identify as Black, 48% single, and 70% publicly insured.^[[3]](#footnote-3)^

**The Original *Preparation for Labor and Birth (P-LAB)* Instrument**

The *P-LAB* is a 22-item instrument used during the third trimester of pregnancy to measure a person’s confidence in their ability to achieve physiologic birth (the spontaneous onset and progression of labor ending in vaginal birth). The initial development and testing of an instrument to measure a person’s broad beliefs about birth, past experiences, knowledge/information, and confidence in their own innate ability to birth. The final conceptual model of childbirth confidence included four interrelated factors: Planned Use of Pain Medication; Relationship With Care Provider and Supportive Birth Environment; Beliefs About Labor; and Labor Support. In its original design, participants answer each question using a 5-point Likert scale ranging from Strongly Disagree to Strongly Agree with items 4, 6, 9, 11, and 20 reverse scored.

**Future Use of the Adapted P-Lab**

We anticipate the final tool will be used to inform strategies that improve prenatal confidence. Additionally, the development of a valid tool for the demographics described above would facilitate further study among marginalized groups, who may have different needs and preferences.

**Key Stakeholder Reviewer Role**

You have been invited to participate because you are recognized by your peers as a leader and/or advocate with knowledge and/or experience regarding community-based birth as well as Black maternal health. Your expertise may be from your lived experiences, professional experiences, training, or a combination of these and other experiences. We have invited a mix of practicing midwives, reproductive equity researchers, potential respondents, and other relevant stakeholders. We greatly value your time and perspective.

**Expectations and Instructions**

As a reviewer, we hope you will complete this written assessment of the tool’s relevance and completeness.

- Please use the response options below to make a judgment about the existing items for measuring childbirth confidence for community-based birth and among CHOICES’ clients.
- For each question think about its relevance to measuring childbirth confidence and rank it on a scale of 0 to 3. For each item, select if “0” you think the item is not relevant, “1” if it is only somewhat relevant, “2” if it is quite relevant, and “3 if it is highly relevant”.
- We invite you to add comments throughout, particularly to support our future group discussion.
- At the end, we also ask about whether you believe the existing items comprehensively measure childbirth confidence; keep this in mind as you go through the questions or navigate back and forth to take additional notes in that space.

After your e-mailed submission of your completed written review, you will receive an invitation to a group discussion for a later date. During this virtual meeting, we will review the collective results from all key stakeholders and collaborate to adapt a tool for piloting with CHOICES’ patients.

*Please continue to the next pages and complete the written assessment.*

**Written Assessment**

| **P-lab Content** | | | | **Key Stakeholder Review Responses** | | |
| --- | --- | --- | --- | --- | --- | --- |
| ***Factor*** | ***Item #*** | ***Prompt*** | ***Response Options*** | ***Relevance to confidence for community-based birth*** | ***Relevance to pregnant care seekers at CHOICES*** | ***Comments*** |
|  |  |  |  | Select the number corresponding to your response for each column   1. Not relevant 2. Somewhat relevant 3. Quite Relevant 4. Highly relevant | |  |
| *Labor support* | 1 | I feel comfortable with where I will give birth | 1. Strongly Disagree 2. Disagree 3. Neutral 4. Agree 5. Strongly Agree | 0  1  2  3 | 0  1  2  3 |  |
| *Labor support* | 2 | I will have the support that I need from my partner, doula, or other support person(s) in labor | 1. Strongly Disagree 2. Disagree 3. Neutral 4. Agree 5. Strongly Agree | 0  1  2  3 | 0  1  2  3 |  |
| *Beliefs about labor* | 3 | I am confident in my body’s ability to labor and birth | 1. Strongly Disagree 2. Disagree 3. Neutral 4. Agree 5. Strongly Agree | 0  1  2  3 | 0  1  2  3 |  |
| *Planned use of medication* | 4 | It is important to me to use medication in labor for pain relief (for example: IV medications or epidural anesthesia) | 1. Strongly Disagree 2. Disagree 3. Neutral 4. Agree 5. Strongly Agree | 0  1  2  3 | 0  1  2  3 |  |
| *Labor support* | 5 | I trust that my prenatal care provider(s) will respect my preferences in labor | 1. Strongly Disagree 2. Disagree 3. Neutral 4. Agree 5. Strongly Agree | 0  1  2  3 | 0  1  2  3 |  |
| *Beliefs about labor* | 6 | When I think about labor and birth, I am fearful | 1. Strongly Disagree 2. Disagree 3. Neutral 4. Agree 5. Strongly Agree | 0  1  2  3 | 0  1  2  3 |  |
| *Labor support* | 7 | I have sought out childbirth information from multiple sources | 1. Strongly Disagree 2. Disagree 3. Neutral 4. Agree 5. Strongly Agree | 0  1  2  3 | 0  1  2  3 |  |
| *Planned use of medication* | 8 | I feel prepared to give birth without the use of pain medication (for example: IV medications or epidural anesthesia) | 1. Strongly Disagree 2. Disagree 3. Neutral 4. Agree 5. Strongly Agree | 0  1  2  3 | 0  1  2  3 |  |
| *Beliefs about labor* | 9 | Negative birth stories from others have made me more fearful about birth | 1. Strongly Disagree 2. Disagree 3. Neutral 4. Agree 5. Strongly Agree | 0  1  2  3 | 0  1  2  3 |  |
| *Beliefs about labor* | 10 | I am excited about experiencing childbirth | 1. Strongly Disagree 2. Disagree 3. Neutral 4. Agree 5. Strongly Agree | 0  1  2  3 | 0  1  2  3 |  |
| *Planned use of medication* | 11 | I plan to give birth with the use of pain medication (for example: I.V. pain medication or epidural anesthesia) | 1. Strongly Disagree 2. Disagree 3. Neutral 4. Agree 5. Strongly Agree | 0  1  2  3 | 0  1  2  3 |  |
| *Labor support* | 12 | My support person(s) (partner, doula, or other) is/are supportive of my childbirth preferences | 1. Strongly Disagree 2. Disagree 3. Neutral 4. Agree 5. Strongly Agree | 0  1  2  3 | 0  1  2  3 |  |
| *Planned use of medication* | 13 | It is important to me to experience childbirth without any pain medication (for example: I.V. pain medication or epidural anesthesia) | 1. Strongly Disagree 2. Disagree 3. Neutral 4. Agree 5. Strongly Agree | 0  1  2  3 | 0  1  2  3 |  |
| *Relationship with care provider and supportive birth environment* | 14 | My prenatal care provider(s) discuss(es) options and choices with me | 1. Strongly Disagree 2. Disagree 3. Neutral 4. Agree 5. Strongly Agree | 0  1  2  3 | 0  1  2  3 |  |
| *Relationship with care provider and supportive birth environment* | 15 | I do not feel that I have enough information about the childbirth process | 1. Strongly Disagree 2. Disagree 3. Neutral 4. Agree 5. Strongly Agree | 0  1  2  3 | 0  1  2  3 |  |
| *Relationship with care provider and supportive birth environment* | 16 | My prenatal care provider communicates with me in an honest and respectful manner | 1. Strongly Disagree 2. Disagree 3. Neutral 4. Agree 5. Strongly Agree | 0  1  2  3 | 0  1  2  3 |  |
| *Labor support* | 17 | I am receiving the right amount of emotional support from my partner, doula, or other labor support person(s) | 1. Strongly Disagree 2. Disagree 3. Neutral 4. Agree 5. Strongly Agree | 0  1  2  3 | 0  1  2  3 |  |
| *Beliefs about labor* | 18 | I am confident that I will be able to cope with labor pain | 1. Strongly Disagree 2. Disagree 3. Neutral 4. Agree 5. Strongly Agree | 0  1  2  3 | 0  1  2  3 |  |
| *Relationship with care provider and supportive birth environment* | 19 | I know my own preferences for labor and birth | 1. Strongly Disagree 2. Disagree 3. Neutral 4. Agree 5. Strongly Agree | 0  1  2  3 | 0  1  2  3 |  |
| *Beliefs about labor* | 20 | I wish I were better prepared for labor and birth | 1. Strongly Disagree 2. Disagree 3. Neutral 4. Agree 5. Strongly Agree | 0  1  2  3 | 0  1  2  3 |  |
| *Relationship with care provider and supportive birth environment* | 21 | My prenatal care provider addresses my needs during prenatal visits | 1. Strongly Disagree 2. Disagree 3. Neutral 4. Agree 5. Strongly Agree | 0  1  2  3 | 0  1  2  3 |  |
| *Relationship with care provider and supportive birth environment* | 22 | My birth will take place in a calm, supportive environment | 1. Strongly Disagree 2. Disagree 3. Neutral 4. Agree 5. Strongly Agree | 0  1  2  3 | 0  1  2  3 |  |

|  | **Comments** |
| --- | --- |
| **Do you believe that, collectively, the items above can comprehensively measure childbirth confidence in a Black-led community setting? Why or why not?** |  |
| **What additional questions, topics, or concepts would you suggest adding?** |  |

**Supporting Appendix S2: Key Stakeholder Group Review Session Guide**

**Preparation for Labor and Birth (P-Lab) Instrument Adaptation**

*Key Stakeholder Discussion Guide*

*Open Zoom Meeting*

**Introduction**

Hi Everyone! Thanks again for your recent participation in the *Key Stakeholder Review* and for your willingness to participate in this follow-up discussion.

We’ll use this time to review the collective results from all key stakeholders and collaborate to adapt a tool for piloting with CHOICES’ patients.

Dr. Nikia Grayson, Dr. Alexis Dunn Amore, and I (Ashley Mitchell) compiled the responses of all Review participants. We have some ideas about adaptations for the P-Lab before we bring it to CHOICES’ patients for cognitive interviewing and, finally, implementation. Still, we want to get your input and ideas based on your experience and expertise.

**Agenda**

Over the next hour we will review a summary of the responses from all stakeholders and discuss items that were neither unanimously “not relevant” nor “highly relevant”. We’ll also open up a dialogue about any comments that were added for particular prompts as well as responses to the final two questions:

- *Do you believe that, collectively, the items above can comprehensively measure childbirth confidence in a Black-led community setting? Why or why not?*
- *What additional questions, topics, or concepts would you suggest adding?*

Finally, we’ll workshop things together toward agreeing upon a final version of an adapted survey. Once adapted, the new survey will be piloted among a small group of CHOICES prenatal patients. We will use cognitive interviewing techniques to assess whether the prompts perform consistently across persons and that they seem to measure what we hope.

Are there any initial questions before we begin?

As mentioned in the consent form, we wish to record this session to ensure that we don’t miss anything and can refer to our conversation until the tool has been fully adapted. Unless there are any objections, we’ll begin recording now. The recording will be stored on the secured and dual password protected server at UCSF which is only accessible to key research team members. It will be deleted upon implementation of the final adaptation of our survey (after any revisions resulting from cognitive interviews).

*Record*

We’ve started the recording.

You may recall that you reviewed a total of 22 existing prompts and that we asked you to rate their relevance for 1) confidence for community-based birth; and 2) pregnant care seekers at CHOICES.

Here are the prompts that reviewers collectively agreed were “not relevant” or only “somewhat relevant”.

*[Display prompts and de-identified reviewer comments]*

1. Is there anything surprising to you among these prompts?
2. Would you advocate that some or all of these be removed for the adapted survey? Why or why not?

Here are the prompts that reviewers generally agreed were “quite relevant” or “highly relevant”.

*[Display prompts and de-identified reviewer comments]*

1. Is there anything to you among these prompts?
2. Do you still agree that these prompts are relevant?
3. Do you all still agree that these prompts should likely be retained in the adapted version of the survey? Why or why not?

Finally, we’ll take a brief look at the responses to the questions that were at the end of the written review before more freely dialoging toward creating a final adaptation.

*[Display list of de-identified reviewer responses to the question “Do you believe that, collectively, the items above can comprehensively measure childbirth confidence in a Black-led community setting? Why or why not?”]*

1. What stands out to you upon reviewing these responses collectively?
2. In your opinion, how does the summary of these responses impact how the survey will need to be adapted for use at CHOICES?

*[Display list of de-identified reviewer responses to the question “What additional questions, topics, or concepts would you suggest adding?”]*

1. What stands out to you upon reviewing these responses collectively?
2. In your opinion, how does the summary of these responses impact how the survey will need to be adapted for use at CHOICES?

*[Stop display]*

1. Having now re-reviewed everything as a group, are there other additional questions, topics, or concepts that come to mind that should be considered for inclusion or exclusion from the adapted survey? Please describe your thought processes.
2. Is there anything that anyone would like to re-review or reconsider together before we work together to produce a final adaptation?

*[Display Word document of the 22 original prompts and continue screen-sharing while edits are made in real-time.]*

The remainder of our meeting will be unstructured conversation with the goal of co-producing an adapted version of the P-lab that feels complete and relevant. I (Ashley) will make edits in real-time as we propose changes or additions together. In areas where we don’t find complete agreement, we’ll rely on input from Nikia and Alexis who have close ties to CHOICES patients.

*[Unstructured dialogue and revising of P-lab document…]*

To wrap up our time together, we’d like to thank you again for your additional time and expertise. We trust your perspectives have made this better and recognize that this research is stronger because of your willingness to help.

If you would like to be added to a dissemination listserv for when our findings our available in the future, please let us know now or send an email to me (Ashley) to let me know.

Take care!

*End Recording*

**
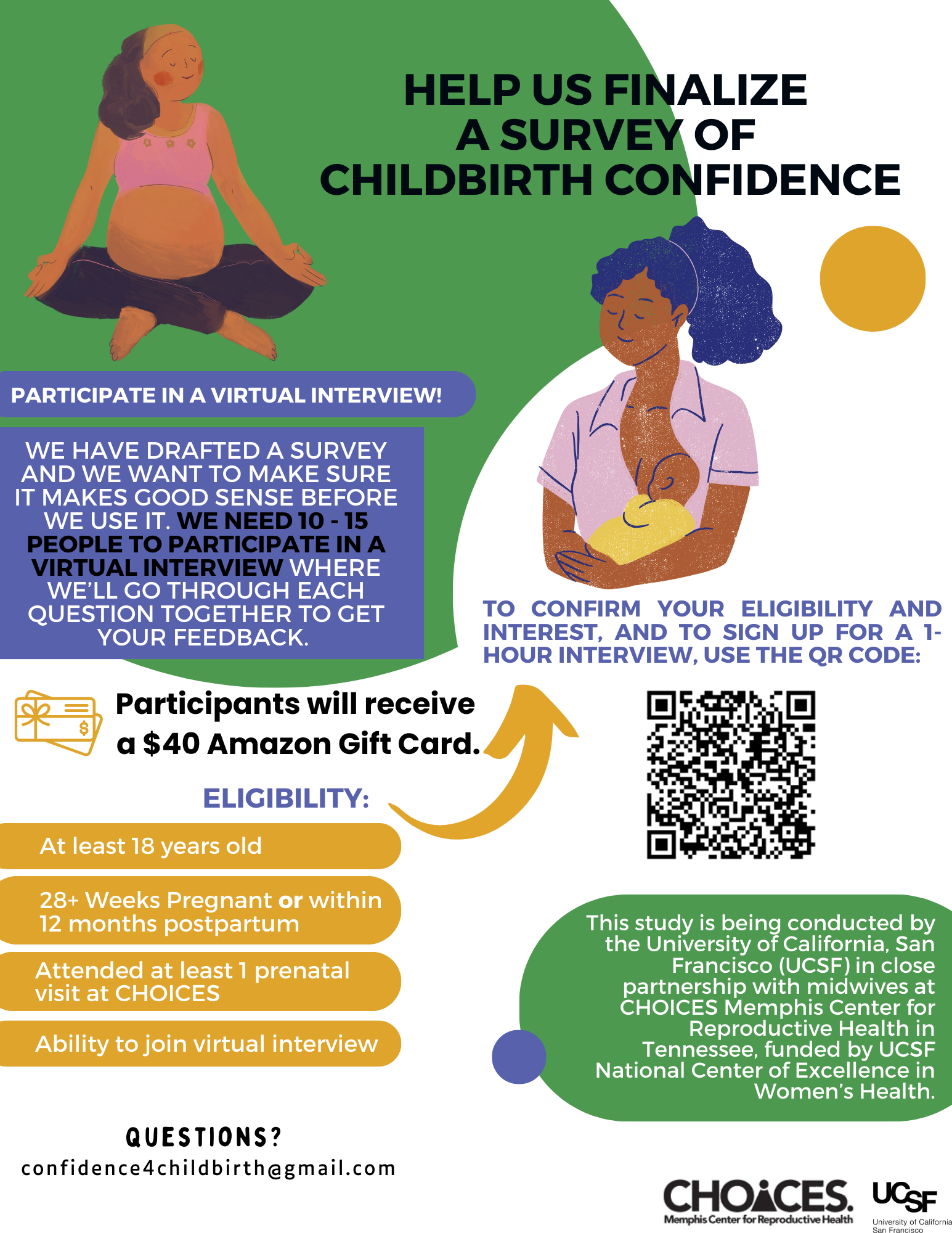
**

**Supporting Figure S1: Recruitment Flyer for Cognitive Interviews**

**Supporting Appendix S3: Cognitive Interview Guide**

**Cognitive Interview Guide and Procedures**

Intro

Hi, I’m Ashley. I’ve been working closely with the midwives at CHOICES. They are interested to know what influences prenatal confidence, so we’re working together to create a tool that measures that. Thanks for your willingness to participate today!

Orientation

Today we’ll spend our time going through the drafted prompts of the survey we adapted to measure prenatal confidence. I will show the prompts on the screen one-by-one. There are 22 short prompts in total. For some of them, I’ll have questions about how you interpret specific concepts. For others, I’ll simply ask you to share what you think.

As shared in the consent form you reviewed while signing up, I would like to record our chat. This is so I can remember all of your ideas to incorporate them later. I’ll be talking to 10 to 15 people total. I hope to see whether different people interpret or react to prompts differently. Then, with the midwives at CHOICES, we can make changes to improve the survey before it is fully implemented.

I have just a couple more details before we begin. I want to remind you that in the future we intend to use the survey with pregnant individuals who are seeking care at CHOICES and places similar to it. This might include other community-based birth centers or places that are equipped to support home births. We are also interested in creating a tool that centers Black birthing persons, like CHOICES does.

Do you have any questions before we get started with verbal consent and then the first prompt?

Is it okay for me to start recording? Yes or No?

***RECORD***

Consent

Thanks for reviewing the consent form sent ahead of our meeting.

- Do you have any questions about it?
- Do you verbally consent to participating in this interview? Remember that you can skip any question you wish and you can stop the interview at any time.

Survey Review

**show prompt 1 with response options*: I feel comfortable with planning to birth in a community setting (birth center, home, etc.)*

**Probe:** What does the term ‘comfortable’ mean to you when you read this prompt?

| **Notes** |
| --- |
|  |

**show prompt 2 with response options:* *I trust that my prenatal care provider(s) will respect my preferences in labor*)*

**Probe:** What does the term ‘respect’ mean to you here?

**Probe:** What types of ‘preferences’ might respondents have in their third trimester of pregnancy?

| **Notes** |
| --- |
|  |

**show prompt 3 with response options:* *I am confident in my body’s ability to labor and birth**

**Probe:** What do you think of this prompt?

**Probe:** Which response item might you select?

**Probe:** Tell me more about how you chose your answer?

| **Notes** |
| --- |
|  |

**show prompt 4 with response options:* *I have been able to find childbirth information that aligned with my priorities (i.e. cultural relevance, trusted source, accessibility, etc.)**

**Probe:** How relevant is this question to your experiences?

**Probe:** What do you think of this prompt?

| **Notes** |
| --- |
|  |

**show prompt 5 with response options:* *I believe that I will have what I need to cope with the pain of labor and birth (i.e. medication, aromatherapy, etc.)**

**Probe:** Which response item might you select?

**Probe:** Tell me more about how you chose your answer?

**Probe:** What do you think of this prompt?

| **Notes** |
| --- |
|  |

**show prompt 6 with response options:* *My prenatal care provider(s) discuss(es) options and choices with me* *

**Probe:** How relevant is this question to your experiences?

**Probe:** What types of “options” and “choices” come to mind when you review this question?

| **Notes** |
| --- |
|  |

**show prompt 7 with response options:* *At this point when I think about labor and birth, I am fearful* *

**Probe:** What do you think of this prompt?

**Probe:** For you, does *fear* feel opposite of *confidence*? Why or why not?

| **Notes** |
| --- |
|  |

**show prompt 8 with response options:* *Negative comments about my birth plan and/or stories from others have made me more fearful* *

**Probe:** How relevant is this question to your experiences?

**Probe:** What do you think of this prompt?

| **Notes** |
| --- |
|  |

**show prompt 9 with response options:* *My birth will take place in a calm, supportive environment* *

**Probe:** How would you define “calm and supportive” in this context?

| **Notes** |
| --- |
|  |

**show prompt 10 with response options:* *My prenatal care provider communicates with me in an honest and respectful manner* *

**Probe:** Which response item might you select?

**Probe:** Tell me more about how you chose your answer?

**Probe:** How do you know when a provider is being *honest and respectful*?

| **Notes** |
| --- |
|  |

**show prompt 11 with response options:* *I’m looking forward to experiencing childbirth* *

**Probe:** Which response item might you select?

**Probe:** Tell me more about how you chose your answer?

**Probe:** What do you think of this prompt?

| **Notes** |
| --- |
|  |

**show prompt 12 with response options:* *Positive comments about my birth plan and/or stories from others have inspired confidence for me* *

**Probe:** How relevant is this question to your experiences?

**Probe:** What do you think of this prompt?

| **Notes** |
| --- |
|  |

**show prompt 13 with response options:* *Given my racial identity, I feel comfortable with the place and providers where I receive prenatal care**

**Probe:** Which response item might you select?

**Probe:** Tell me more about how you chose your answer?

**Probe:** Do you think this question gets at the provision of antiracist care? Why or why not?

| **Notes** |
| --- |
|  |

**show prompt 14 with response options:* *My prenatal care provider addresses my needs during prenatal visits**

**Probe:** What types of *needs* come to mind when you review this question?

| **Notes** |
| --- |
|  |

**show prompt 15 with response options:* *I know my own preferences for labor and birth**

**Probe:** Which response item might you select?

**Probe:** Tell me more about how you chose your answer?

**Probe:** How relevant is your knowledge and preferences to your perception of how confident you feel?

| **Notes** |
| --- |
|  |

**show prompt 16 with response options:* *When I visualize the birth that I want, I have the tools that I need to accomplish it (i.e. mindfulness, meditation, spirituality)—or I know where to find them by the time I birth**

**Probe:** How relevant is this question to your experiences?

**Probe:** What do you think of this prompt?

| **Notes** |
| --- |
|  |

**show prompt 17 with response options:* *I feel safe in the physical space that I plan to birth**

**Probe:** How would you define *safe* in the context of this prompt?

| **Notes** |
| --- |
|  |

**show prompt 18 with response options:* *I feel protected by my prenatal provider* *

**Probe:** Which response item might you select?

**Probe:** Tell me more about how you chose your answer?

**Probe:** How would you define *protected* in the context of this prompt?

| **Notes** |
| --- |
|  |

**show prompt 19 with response options:* *My self-talk as I prepare for birth is mostly positive**

**Probe:** Which response item might you select?

**Probe:** Tell me more about how you chose your answer?

**Probe:** How does/did self-talk relate to confidence in your experience?

| **Notes** |
| --- |
|  |

**show prompt 20 with response options:* *I wish I were better prepared for labor and birth**

**Probe:** How relevant is this question to your experiences?

**Probe:** What is entailed with *preparation* in this context, from your experience? Does that relate to confidence?

| **Notes** |
| --- |
|  |

**show prompt 21 with response options:* *I sense that my prenatal provider sees, knows, and cares about me as a person—or will make every effort to do so by the time I birth**

**Probe:** Which response item might you select?

**Probe:** Tell me more about how you chose your answer?

| **Notes** |
| --- |
|  |

**show prompt 22 with response options:* *I believe I will have the support that I need from my partner, doula, or other non-medical support person(s) in labor**

**Probe:** How relevant is this question to your experiences?

**Probe:** What do you think of this prompt?

| **Notes** |
| --- |
|  |

**show just the response options:* *0. Strongly Disagree, 1. Disagree, 2. Neutral, 3. Agree, 4. Strongly Agree**

**Probe:** The final detail that we have discussed is whether or not to include “Neutral” as a response option across all questions. What are your thoughts about this response option?

**Probe:** In what cases do you feel a “Neutral” option would be beneficial or necessary?

| **Notes** |
| --- |
|  |

**show stop screen-sharing**

Closing Thoughts

We have reviewed all the prompts and details of the current survey—THANK YOU! Now, I am open to hearing any additional thoughts or feedback you have.

**Probe:** Is there anything else you think contributes to childbirth confidence that we haven’t considered?

**Probe:** Are there any topics that we covered too much?

**Probe:** Is there anything else you want to share related to the topics we’ve covered today?

Thanks

Thank you so much for your participation today. I’ll stop the recording now.

***STOP RECORDING***

You will receive the e-gift card via the e-mail you used to sign-up for this interview. Please reach out via email if you have any questions or anything after we log off. I am grateful for your time, take care.


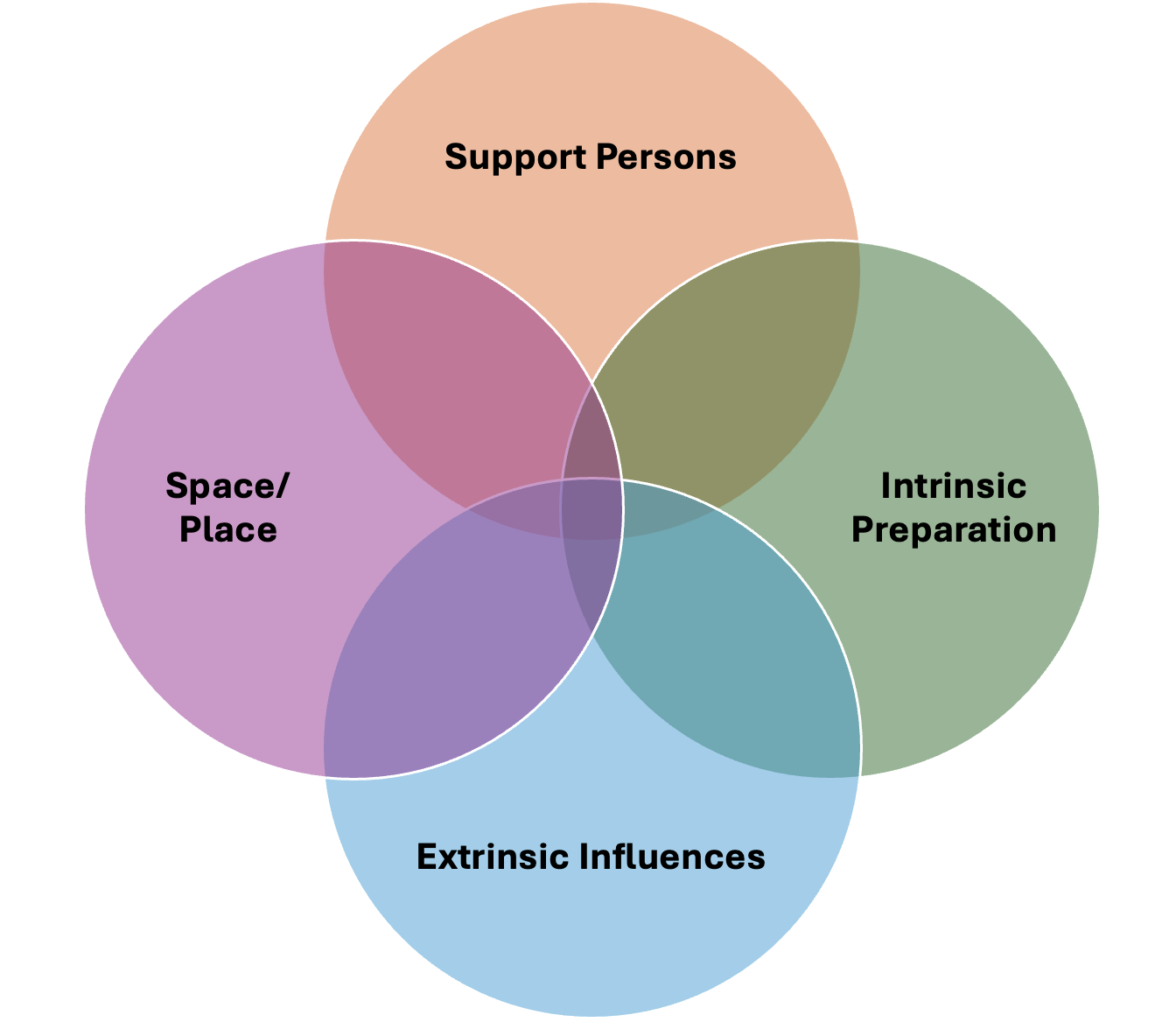


**Supporting Figure S2: Adapted Conceptual Model of Confidence for Community-Based Birth**

**Supporting Table S1: Cognitive Interview Findings Summaries Mapped By A Priori Themes**

| **Cognitive interview findings summaries mapped by theme** | | | | |
| --- | --- | --- | --- | --- |
| **#** | **Prompt** | **Compression (meaning of words)** | **Judgement (Ease of answering)** | **Perceived relation to Confidence** |
| 1 | I feel comfortable with planning to birth in a community setting (birth center, home, etc.) | “I felt at home” | One person got stuck on the fact that they wouldn't at home, but they would at a birthing center | comfort translated to confidence |
| 2 | I trust that my prenatal care provider(s) will respect my preferences in labor | “when they honor what I say what’s best for me"; many described being *really* listened to | N/A, determined to be straightforward | respect translated to confidence |
| 3 | I am confident in my body’s ability to labor and birth | “It’s a mind over matter thing”; several described the midwives/education improving confidence in this | “not strongly because I know everybody’s body is different”; several hesitated as it doesn't capture complexities | N/A, "confident" in prompt |
| 4 | I have been able to find childbirth information that aligned with my priorities (i.e. cultural relevance, trusted source, accessibility, etc.) | “what’s a trusted source?”; several described that if they had questions, they could find answers | several hesitated and got hung up on items in the parentheses | information was "empowering" |
| 5 | I believe that I will have what I need to cope with the pain of labor and birth (i.e. medication, aromatherapy, etc.) | "they made sure I had what I needed before I gave birth"; examples like water, music, breathing, comb, ball | Medication was a stumbling point as most thought of epidural | believing they could achieve some level of comfort translated to confidence |
| 6 | My prenatal care provider(s) discuss(es) options and choices with me | “everything was my choice, I always had a choice”; examples: use of water, nutrition in pregnancy | Several folks got stuck because they weren't sure what this was asking | One person suggested that getting info (and consent forms) ahead of time helped their confidence |
| 7 | At this point when I think about labor and birth, I am fearful | understanding that “you can conquer fear" with the right support | many processed fear of pain/undesirable outcomes yet confidence because of preparation | Most perceived you can be *both* fearful and confident |
| 8 | Negative comments about my birth plan and/or stories from others have made me more fearful | most jumped to each person having a unique experience, “Their story it doesn’t have to be my story” | two people wondered if this should be specific to community birth | No indication of irrelevance |
| 9 | My birth will take place in a calm, supportive environment | "a space where my mind was is at ease and I can focus on labor” | N/A, determined to be straightforward | Calm support translated to confidence |
| 10 | My prenatal care provider communicates with me in an honest and respectful manner | “I feel like a person and that’s really important for a pregnant person to feel like a person”; examples of being cared for and treated as an equal | many explained this increased trust | honesty and respect translated to confidence |
| 11 | I’m looking forward to experiencing childbirth | “I’m more looking forward to proving to myself that I can do it”; many also described being ready to not be pregnant and/or meet their baby | influenced by prior birth experiences for folks with kids | No indication of irrelevance |
| 12 | Positive comments about my birth plan and/or stories from others have inspired confidence for me | “It makes me feel like I’m doing the right thing for my baby and my body” | Several people brought up their own past birth experiences | positivity translated to confidence |
| 13 | Given my racial identity, I feel comfortable with the place and providers where I receive prenatal care | “I see me”; many described the benefits of racial concordance | one white person conceptualized about guilt | concordance translated to confidence |
| 14 | My prenatal care provider addresses my needs during prenatal visits | described midwives proactively anticipating and addressing physical and mental needs | N/A, determined to be straightforward | ”when I come to this visit, I don’t need to be an expert in this field”; increased confidence |
| 15 | I know my own preferences for labor and birth | A couple participants specifically referenced birth plans | One person said they prefer to "go with the flow" and that made this hard to answer | “because I feel like I had something to go off of during the process… I didn’t have to think on the fly”; increased confidence |
| 16 | When I visualize the birth that I want, I have the tools that I need to accomplish it (i.e. mindfulness, meditation, spirituality)—or I know where to find them by the time I birth | many described music, dim lights, oils, water, massage, positive self talk | several hesitated or needed clarity and got lost in the length of the prompt | No indication of irrelevance |
| 17 | I feel safe in the physical space that I plan to birth | “my mental and physical being are 100% being looked after” | N/A, determined to be straightforward | safety translated to confidence |
| 18 | I feel protected by my prenatal provider | “If there was anything that was going to cause me any harm …I very much so had midwives who were like *‘I’m going to be there for you’*.” | N/A, determined to be straightforward | protection translated to confidence |
| 19 | My self-talk as I prepare for birth is mostly positive | “I believe that what you speak will manifest” | N/A, determined to be straightforward | one person said it was potentially unrelated, for others it improved confidence |
| 20 | I wish I were better prepared for labor and birth | "having a plan"; some discussed classes or having their birth bag ready | “I’m still preparing”; several commented on things they planned to do still that influenced how they would answer | preparation translated to confidence |
| 21 | I sense that my prenatal provider sees, knows, and cares about me as a person—or will make every effort to do so by the time I birth | “I wasn’t just a number or checking things off of a list” | N/A, determined to be straightforward | person-centered care translated to confidence |
| 22 | I believe I will have the support that I need from my partner, doula, or other non-medical support person(s) in labor | examples included doula, partner, parent, child, etc. | many still included midwives/medical support people when conceptualizing | “boosted it up 1000%” |

**Supporting Table S2: Instrument Item Evolution from Original Items to Final Adapted Item Following Expert and Community Stakeholder Review Processes**

| # | **Factor** | **Original P-LAB Item** | **Post-Expert Review** | **Post-Community Review** | **Factor** | **#** |
| --- | --- | --- | --- | --- | --- | --- |
| 1 | Labor support | I feel comfortable with where I will give birth | I feel comfortable with planning to birth in a community setting (birth center, home, etc.) | I feel comfortable with planning to birth in a community setting (i.e. not at a hospital) | Space/ Place | 1 |
| 2 | Labor support | I will have the support that I need from my partner, doula, or other support person(s) in labor | I believe I will have the support that I need from my partner, doula, or other non-medical support person(s) in labor | I believe I will have the support that I need from my partner, doula, and/or other non-medical support person(s) in labor | Support Persons | 2 |
| 3 | Beliefs about labor | I am confident in my body’s ability to labor and birth | I am confident in my body’s ability to labor and birth | I am confident in my body’s ability to labor and birth | Intrinsic Preparation | 3 |
| 4 | Planned use of medication | It is important to me to use medication in labor for pain relief (for example: IV medications or epidural anesthesia) |  |  |  |  |
| 5 | Labor support | I trust that my prenatal care provider(s) will respect my preferences in labor | I trust that my prenatal care provider(s) will respect my preferences in labor | I trust that my prenatal care provider(s) will respect my preferences in labor | Support Persons | 4 |
| 6 | Beliefs about labor | When I think about labor and birth, I am fearful | At this point when I think about labor and birth, I am fearful | At this point when I think about labor and birth, I am more confident than fearful | Intrinsic Preparation | 5 |
| 7 | Labor support | I have sought out childbirth information from multiple sources | I have been able to find childbirth information that aligned with my priorities (i.e. cultural relevance, trusted source, accessibility, etc.) | When I have had questions, I have been able to find childbirth information that I found beneficial | Extrinsic Influences | 6 |
| 8 | Planned use of medication | I feel prepared to give birth without the use of pain medication (for example: IV medications or epidural anesthesia) |  |  |  |  |
| 9 | Beliefs about labor | Negative birth stories from others have made me more fearful about birth | Negative comments about my birth plan and/or stories from others have made me more fearful | Negative comments about my birth plan, my own past experiences, and/or stories from others have made me more fearful | Extrinsic Influences | 7 |
| 10 | Beliefs about labor | I am excited about experiencing childbirth | I’m looking forward to experiencing childbirth | I feel ready for birth (or believe I will be by the time the baby comes) | Intrinsic Preparation | 8 |
| 11 | Planed use of medication | I plan to give birth with the use of pain medication (for example: I.V. pain medication or epidural anesthesia) |  |  |  |  |
| 12 | Labor support | My support person(s) (partner, doula, or other) is/are supportive of my childbirth preferences | Positive comments about my birth plan and/or stories from others have inspired confidence for me | Positive comments about my birth plan, my own past experiences, and/or stories from others have inspired confidence for me | Extrinsic Influences | 9 |
| 13 | Planned use of medication | It is important to me to experience childbirth without any pain medication (for example: I.V. pain medication or epidural anesthesia) |  |  |  |  |
| 14 | Relationship with care provider and supportive birth environment | My prenatal care provider(s) discuss(es) options and choices with me | My prenatal care provider(s) discuss(es) options and choices with me | My prenatal care provider(s) are giving me what I need to feel prepared for labor and birth (i.e. educating me on my options/choices) | Support Persons | 10 |
| 15 | Relationship with care provider and supportive birth environment | I do not feel that I have enough information about the childbirth process |  |  |  |  |
| 16 | Relationship with care provider and supportive birth environment | My prenatal care provider communicates with me in an honest and respectful manner | My prenatal care provider communicates with me in an honest and respectful manner | My prenatal care provider communicates honestly with me | Support Persons | 11 |
| 17 | Labor support | I am receiving the right amount of emotional support from my partner, doula, or other labor support person(s) |  |  |  |  |
| 18 | Beliefs about labor | I am confident that I will be able to cope with labor pain | I believe that I will have what I need to cope with the pain of labor and birth (i.e. medication, aromatherapy, etc.) | I believe that I will have what I need to cope with the pain of labor and birth (i.e. aromatherapy, a birthing tub, etc.) | Space/ Place | 12 |
| 19 | Relationship with care provider and supportive birth environment | I know my own preferences for labor and birth | I know my own preferences for labor and birth | I know my own preferences for labor and birth—even if my preference is going with the flow | Intrinsic Preparation | 13 |
| 20 | Beliefs about labor | I wish I were better prepared for labor and birth | I wish I were better prepared for labor and birth | I am worried that when labor begins, I won't be prepared for it | Intrinsic Preparation | 14 |
| 21 | Relationship with care provider and supportive birth environment | My prenatal care provider addresses my needs during prenatal visits | My prenatal care provider addresses my needs during prenatal visits | My prenatal care provider addresses my needs during prenatal visits | Support Persons | 15 |
| 22 | Relationship with care provider and supportive birth environment | My birth will take place in a calm, supportive environment | My birth will take place in a calm, supportive environment | My birth will take place in a calm, supportive environment | Space/ Place | 16 |
|  |  |  | *Newly added:* Given my racial identity, I feel comfortable with the place and providers where I receive prenatal care | Given my racial identity, I feel comfortable with the providers with whom I receive prenatal care | Space/ Place | 17 |
|  |  |  | *Newly added:* When I visualize the birth that I want, I have the tools that I need to accomplish it (i.e. mindfulness, meditation, spirituality)—or I know where to find them by the time I birth | When I visualize the birth that I want, I believe I will have the tools that I need to accomplish it (i.e. music, knowledge of different positions to labor/birth, breathing techniques, etc.) | Extrinsic Influences | 18 |
|  |  |  | *Newly added:* I feel safe in the physical space that I plan to birth | I feel safe in the physical space that I plan to birth | Space/ Place | 19 |
|  |  |  | *Newly added:* I feel protected by my prenatal provider | I feel protected by my prenatal provider | Support Persons | 20 |
|  |  |  | *Newly added:* My self-talk as I prepare for birth is mostly positive | My self-talk as I prepare for birth is mostly positive | Intrinsic Preparation | 21 |
|  |  |  | *Newly added:* I sense that my prenatal provider sees, knows, and cares about me as a person—or will make every effort to do so by the time I birth | I sense that my prenatal provider cares about me as a person—or will make every effort to do so by the time I birth | Support Persons | 22 |
|  |  |  |  | *Newly added:* I have people who listen to and believe me about my experiences in pregnancy | Support Persons | 23 |

1. Thayer ZM, Geisel-Zamora SA, Uwizeye G, Gildner TE. Childbirth fear in the USA during the COVID-19 pandemic: key predictors and associated birth outcomes. Evol Med Public Health 2023; 11: 101–11. [↑](#footnote-ref-1)
2. Neerland CE, Avery MD, Looman WS, Saftner MA, Rockwood TH, Gurvich OV. Development and Testing of the Preparation for Labor and Birth Instrument. J Obstet Gynecol Neonatal Nurs JOGNN 2020; 49: 200–11. [↑](#footnote-ref-2)
3. Dunn Amore A, Quinones N, Mitchell A, Blair A, Grayson N. Accepted & Under Review: Unveiling the Transformative Impact of the CHOICES Community-Based Model on Birthing Parents Outcomes. 2023. [↑](#footnote-ref-3)
