# Supplementary material for: Development of the Preparation for Community‐Based Labor and Birth Instrument Centering Black Perspectives in the United States: A Participatory Adaptation
Source: J Midwifery Womens Health. 2025 Oct 28;71(1):76–86. doi: 10.1111/jmwh.70040 (PMC12914615; doi:10.1111/jmwh.70040)
Supplement: Supplementary file 2 — Supporting Information [file JMWH-71-76-s001.docx]

For the adaptation process, we relied on the first seven Guidelines in Scale Development outlined in Chapter 5 of *Scale Development: Theory and Applications (5^th^ Edition)*.^1^ This is referenced in the first sentence of our Methods section. The steps and where we describe them in our manuscript are as follows:

| **Step** | **Where described in manuscript** |
| --- | --- |
| 1: Determine Clearly What It Is You Want to Measure | P 1 “birthing confidence in the third trimester”; Pp 3-4 “A targeted instrument intended for community-based clinical use may allow for more timely evaluation of prenatal interventions intended to improve experiences or confidence. It may also assist with determining the most relevant birth setting for an individual given the differing resources available in a hospital versus community setting. Accordingly, the aim of this project was to adapt the P-LAB instrument for use with community-based care seekers, centering the perspectives of Black birthing people.” |
| 2: Generate Item Pool | Utilized existing 22-item pool, P 4 “We followed standard instrument development guidelines to adapt the 22-item Likert-response P-LAB instrument using an iterative approach involving both an expert and community stakeholder review between April and October 2024.” |
| 3: Determine the Format for Measurement | Utilized existing Likert format, P 4 “We followed standard instrument development guidelines to adapt the 22-item Likert-response P-LAB instrument using an iterative approach involving both an expert and community stakeholder review between April and October 2024.” |
| 4: Have Initial Item Pool Reviewed By Experts | Conducted expert stakeholder review, discussed in Methods pp 4-5 |
| 5: Cognitive Interviewing | Conducted cognitive interviewing as part of community stakeholder review outlined in Methods p 5 |
| 6: Consider Inclusion of Validation Items | Will be described following psychometric evaluation in future publication, p 11 “The P-CLAB is now being administered at multiple, Black-led birth centers in the U.S. and psychometric analysis is planned toward further validation.” |
| 7: Administer Items to a Development Sample | Ongoing, p 11 “The P-CLAB is now being administered at multiple, Black-led birth centers in the U.S. and psychometric analysis is planned toward further validation.” |

**Reference:**

1. DeVellis RF, Thorpe CT. *Scale Development: Theory and Applications*. SAGE Publications; 2021.
